# External validation of the Phoenix Sepsis Score in a paediatric intensive care unit in Saudi Arabia

**DOI:** 10.1038/s41598-026-48807-6

**Published:** 2026-04-14

**Authors:** Yasser Mohammed Kazzaz, Naila Shaheen, Hamad Alkhalaf, Ghaida Mashraqi, Aisha Abdullah Asidan, Rayouf Mohammed Almojel, Nawaf Abdullah Alghamdi, Majd Abdullah Alomar, Hamza Alali

**Affiliations:** 1 Department of Paediatrics, King Abdullah Specialised Children’s Hospital, Ministry of the National Guard-Health Affairs, Riyadh, Saudi Arabia; 2 King Saud bin Abdulaziz University for Health Sciences, Riyadh, Saudi Arabia; 3 King Abdullah International Medical Research Centre, Riyadh, Saudi Arabia; 4 Division of Biostatistics, Department of Population Health, King Abdullah International Medical Research Center, Riyadh, Saudi Arabia

**Keywords:** Sepsis, Septic shock, Paediatric intensive care unit, Criteria, Saudi Arabia, Bacterial infection, Viral infection, Infectious diseases

## Abstract

We externally validated the Phoenix Sepsis Score (PSS) for predicting n-hospital mortality in a Paediatric Intensive Care Unit (PICU) setting in Saudi Arabia and compared its performance with those of the International Paediatric Sepsis Consensus Conference (IPSCC) criteria and Paediatric Logistic Organ Dysfunction-2 (PELOD-2) score. This retrospective cohort study of a prospectively maintained paediatric sepsis registry included 0–14-year-old PICU-admitted children with suspected sepsis between 2015 and 2023. Of 431 admissions, 281 (65.2%) met the PSS criteria (PSS ≥ 2), of whom 197/281 patients (70.1%) met the septic shock criteria. The in-hospital mortality rates were 21.8% for the entire cohort, 12% for infection, and 27% for sepsis. A PSS ≥ 2 yielded a sensitivity of 80.9% (95% confidence interval [CI]: 71.7–87.5) and positive predictive value of 27.0% (95%CI: 22.2–32.5). The area under the precision-recall curve for the PSS was 0.45 (95% CI: 0.35–0.56), which was higher than that for the IPSCC criteria (0.38, 95%CI: 0.29–0.48) and comparable to that for the PELOD-2 score (0.48, 95%CI: 0.37–0.58). PSS discriminative performance, assessed by the area under the receiver-operating characteristic curve, was 0.67 (95% CI: 0.61–0.74), similar to that of the IPSCC criteria (0.67, 95%CI: 0.60–0.73) and PELOD-2 score (0.72, 95%CI: 0.65–0.77). The PSS demonstrated favourable precision-recall performance and fair discrimination for in-hospital mortality in this critical care cohort. Its precision-recall performance was superior to that of the IPSCC criteria and comparable to that of the PELOD-2 score. Notably, the PSS relies on fewer variables across four organ systems, which may enhance its practicality for risk stratification in comparable high-acuity critically ill children.

**Supplementary Information:**

The online version contains supplementary material available at 10.1038/s41598-026-48807-6.

## Introduction

Sepsis remains a serious public health problem, with high morbidity and mortality rates worldwide. A prospective study across 26 countries reported a point prevalence of 8.2% and mortality rate of 25%. The prevalence and mortality rates in paediatric intensive care units (PICUs) vary among different regions, with a prevalence of 15.3% and a mortality rate of 40% in Asia^1^. Several single-centre studies from Saudi Arabia have reported a mortality rate of 16.9–30%^2–5^. Sepsis was the leading cause of death in a single PICU study in Saudi Arabia (31%), followed by respiratory infections (19%)^6^.

The diagnosis of paediatric sepsis was formerly based on the systemic inflammatory response syndrome (SIRS) criteria, established through expert consensus and published by the International Paediatric Sepsis Consensus Conference (IPSCC) in 2005^7^. In 2016, the Third International Consensus Definitions for Sepsis and Septic Shock (Sepsis-3) introduced a revised definition for adult sepsis. This definition was not based on the SIRS criteria but utilised organ dysfunction assessed using the Sequential Organ Failure Assessment Score. Notably, Sepsis-3 differed from prior definitions by being data-driven and developed through the analysis of large patient cohorts from the United States and Germany^8^. Two decades after publishing the first paediatric sepsis criteria, the Society of Critical Care Medicine (SCCM) Paediatric Sepsis Definition Task Force published a new definition of sepsis, replacing SIRS with multi-organ failure assessed by the Phoenix Sepsis Score (PSS). The PSS was developed using a combination of an international clinician survey, a systematic review and meta-analysis, data-driven modelling from a large multinational database that included over 200,000 encounters with patients with infection, and expert consensus through a modified Delphi procedure^9^. The PSS is calculated in children with suspected infection based on the functioning of four organ systems: the cardiovascular, respiratory, coagulation, and neurological systems. A score of ≥ 2 indicates sepsis, and the presence of at least one cardiovascular point signifies septic shock. These criteria are used in clinical decision making, research, epidemiological studies, and quality improvement^10^.

The SCCM Paediatric Sepsis Definition Task Force acknowledged the need for further external validation, noting that the data were predominantly derived from a US cohort (> 80% of the study population), with the remainder coming from four other countries (Bangladesh, Colombia, China, and Kenya)^9^. This underscores the importance of validating the criteria in countries with distinct healthcare systems^11–13^. Saudi Arabia, classified as a high-resource country with an advanced healthcare system, represents one such setting^14^. Given that the Phoenix criteria have not yet been validated in Saudi Arabia, our primary objective was to externally validate the PSS in a cohort of critically ill children admitted to a PICU in Saudi Arabia. The secondary objective was to compare the PSS with the IPSCC criteria and Paediatric Logistic Organ Dysfunction-2 (PELOD-2) score.

## Materials and methods

This retrospective cohort study was conducted at King Abdullah Specialised Children’s Hospital, a tertiary paediatric referral centre in Riyadh, Saudi Arabia. This study utilised data from the Paediatric Sepsis Registry of our PICU, which prospectively included critically ill children aged 0–14 years admitted to the PICU with abnormal temperature or white blood cell count, microbiological cultures obtained, prescribed antimicrobial therapy, and clinically identified sepsis by the treating physician at the time of admission. The sample comprised all consecutive eligible admissions recorded in the registry during the study period. No additional exclusion criteria were applied. Repeated admissions were treated as separate encounters.

The registry and study protocol were approved by the Institutional Review Board of King Abdullah International Medical Research Centre. The registry prospectively captures detailed clinical information including basic demographics, microbiologic test details, therapeutic interventions, and serial physiological and laboratory variables. Daily PELOD-2 scores were recorded for the first 3 days of PICU admission, along with key management data such as mechanical ventilation, vasoactive support, and renal replacement therapy. Phoenix variables were obtained retrospectively from electronic health records through independent manual review of individual patient records, using the most abnormal values within 24 h for score calculation. In our unit, a standardized admission workup, including coagulation parameters is routinely performed for all PICU patients. The PSS was calculated using available clinical and laboratory data in accordance with the original consensus methodology^9^. For this analysis, we included all patients recorded in the registry between May 2015 and December 2023. The PELOD-2 score was calculated using the day-1 score (first 24 h of PICU admission). The IPSCC criteria were operationalized by summing the number of organ dysfunctions to obtain a total organ dysfunction count, as previously described^9^. For the present analysis, all variables required to compute the respective scores were available for included encounters; therefore, no imputation procedures or exclusions due to missing data were applied.

### Statistical analysis

Statistical analysis was conducted using SAS version 9.4 (SAS Institute, Cary, NC, USA). The IPSCC criteria were utilised by converting the number of organ dysfunction counts to obtain a total organ dysfunction count. The performances of the PSS, PELOD-2 score-day 1, and IPSCC organ dysfunction count were evaluated for in-hospital mortality using the area under the precision–recall curve (AUPRC) and the area under the receiver-operating characteristic curve (AUROC), with corresponding 95% confidence intervals. ROC curves were generated for each score, and pairwise comparisons of AUROC values were performed using the nonparametric DeLong test. Comparisons between patients classified as infection and sepsis according to the Phoenix criteria, as well as between survivors and non-survivors, were performed. Comparisons between patients classified as sepsis and septic shock according to the Phoenix criteria were conducted as exploratory subgroup analyses. Categorical variables are presented as frequencies and percentages and were compared using the chi-square or Fisher exact test. Continuous variables are summarised as median/interquartile range (IQR) and compared using the Wilcoxon rank-sum test. Data distribution was examined for normality using the Kolmogorov–Smirnov and Shapiro–Wilk tests. Statistical significance was set at *p* < 0.05.

## Results

A total of 431 PICU admissions meeting the inclusion criteria were analysed. The clinical characteristics of the patients are shown in Table 1. Based on the Phoenix criteria, 150 of 431 cases (34.8%) were classified as infected, while 281 cases (65.2%) met the criteria for sepsis (PSS ≥ 2). Of the 281 patients with sepsis, 197 (70.1%) met the criteria for septic shock, defined as sepsis with a cardiovascular dysfunction score of 1. Patients with sepsis were older (median age: 22 vs. 11 months, *p* = 0.002), had a higher rate of positive bacterial cultures (122/281 [43.4%] vs. 46/150 [30.7%], *p* = 0.013), and had significantly higher severity scores, including the Paediatric Index of Mortality (PIM) 3 (median 9% vs. 2%, *p* < 0.001) and PELOD-2 score on day 1 (median 7 vs. 2.5, *p* < 0.001), than those with infection. In addition, the sepsis group had a longer median PICU length of stay (7 vs. 2.5 days, *p* < 0.001) and higher in-hospital mortality (76/281 [27.0%] vs. 18/150 [12.0%], *p* = < 0.001).

Online Resource 1 shows the clinical characteristics of survivors (*n* = 337) and non-survivors (*n* = 94). Non-survivors had significantly higher PSS (median 4.0 vs. 2.0), PELOD-2 score (8.0 vs. 4.0), and IPSCC count (6.0 vs. 4.0) (*p* < 0.001). The use of inotropes (64.9% vs. 33.2%), intubation (78.7% vs. 34.1%), and continuous renal replacement therapy (12.8% vs. 3.0%) was significantly higher in non-survivors than in survivors (*p* < 0.001). Non-survivors also had more frequent positive bacterial (53.2% vs. 35.0%, *p* = 0.001) and fungal (7.5% vs. 1.8%, *p* = 0.01) results and fewer positive viral results (29.8% vs. 44.8%, *p* = 0.009) than survivors.

Online Resource 2 shows the clinical characteristics of the patients with sepsis and septic shock based on the Phoenix criteria. Children with septic shock were significantly older (median 28.0 vs. 14.0 months, *p* = 0.036), had higher predicted mortality (PIM 3: 10% vs. 7%, *p* = 0.033), and had greater organ dysfunction (PELOD-2: 8.18 vs. 5.85, *p* < 0.001) than those with only sepsis. The sepsis group had longer PICU (median 13.3 vs. 8.9 days, *p* < 0.001) and hospital stays (60.1 vs. 54.7 days, *p* < 0.001) than the septic shock group. The mortality rates were not significantly different between the two groups (23.8% vs. 28.4%, *p* = 0.425). The shorter length of stay observed in the septic shock group was likely attributable to earlier mortality despite intensive support. In contrast, children with sepsis who died tended to have more prolonged courses, with mortality due to complications such as hospital-acquired infections, progressive multiorgan dysfunction, late goals of care, and limitations of life-sustaining therapies. Subgroup analysis confirmed this finding, showing that septic shock non-survivors had a significantly shorter PICU stay (median 5 days, IQR: 1.75–13) than sepsis non-survivors (median 10 days, IQR: 5.75–47; *p* = 0.003).

**Fig. 1:**
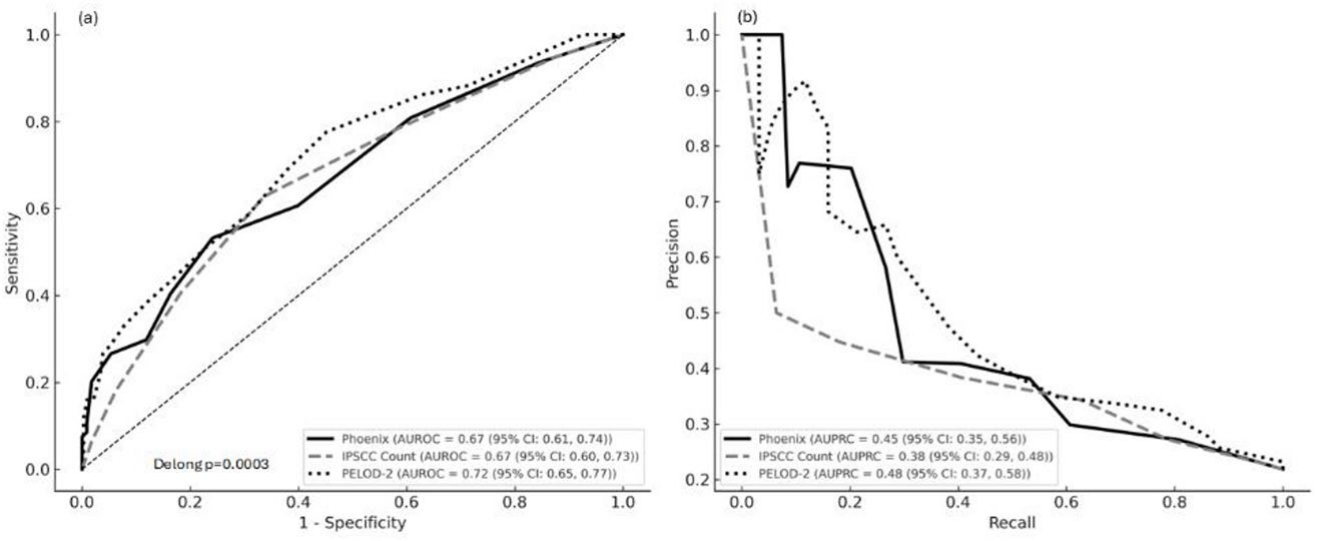
Receiver-operating characteristic (ROC) curves and precision-recall (PR) curves for Phoenix Sepsis Scores (PSS), Paediatric Logistic Organ Dysfunction-2 (PELOD-2) score and International Paediatric Sepsis Consensus Conference (IPSCC) Criteria. CI, confidence interval; AUPRC, area under the precision-recall curve; AUROC, area under the ROC curve.

**Fig. 2:**
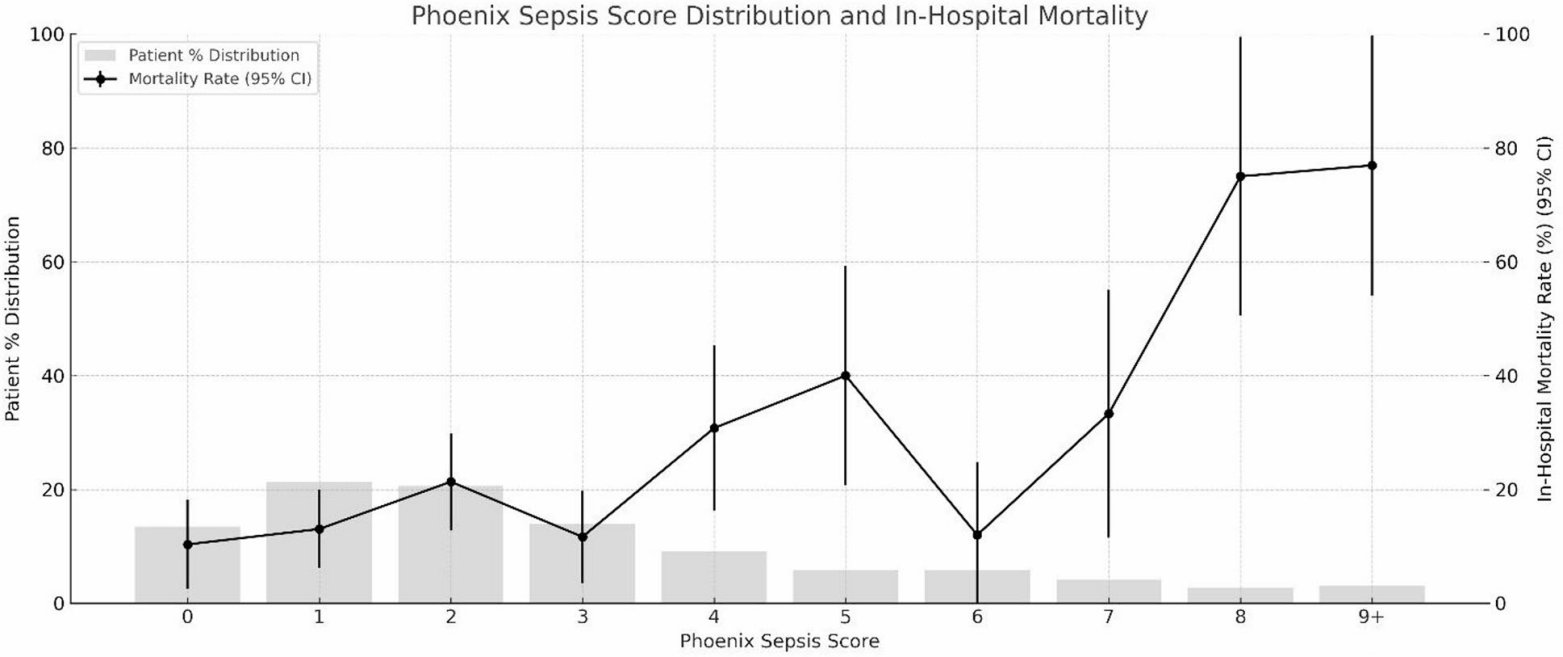
Distribution of Phoenix Sepsis Score and associated in-hospital mortality in critically ill children admitted with confirmed or suspected infection. CI, confidence interval.

For in-hospital mortality prediction, a PSS ≥ 2 demonstrated a sensitivity of 80.9% (95% confidence interval [CI]: 71.7%–87.5%) and a positive predictive value (PPV) of 27.0% (95% CI: 22.2%–32.5%). Table 2; Fig. 1 present the AUPRC and AUROC values for the PSS, PELOD-2 score, and IPSCC count. The AUPRC for the PSS was 0.45 (95% CI: 0.35–0.56), which was higher than the IPSCC count (0.38, 95% CI: 0.29–0.48) and comparable to the PELOD-2 score (0.48, 95% CI: 0.37–0.58). For discrimination, the AUROC for the PSS was 0.67 (95% CI: 0.61–0.74), similar to the IPSCC count (0.67, 95% CI: 0.60–0.73) and comparable to the PELOD-2 score (0.72, 95% CI: 0.65–0.77). Overall comparison of ROC curves using the nonparametric DeLong test indicated a statistically significant difference among the three scores (*p* = 0.0003), although pairwise comparisons did not show significant differences between all score combinations. Figure 2 presents the association between in-hospital mortality and the PSS, demonstrating a proportionate increase in mortality with increasing scores.

**Table 1:** Clinical characteristics of critically ill children with suspected sepsis admitted to the PICU: Comparison of infection vs. sepsis defined by the Phoenix criteria.

Variable	Total cohort (*N* = 431)	Infection (No sepsis) (*N* = 150)	Sepsis (*N* = 281)	*p*-value
Male	238 (55.2%)	86 (57.3%)	152 (54.1%)	0.519
Age (months)	22 (11–89)	11 (2.5–33)	22 (7–89)	0.002
Any comorbidities	334 (77.5%)	116 (77.3%)	218 (77.6%)	0.953
Prematurity	69 (16.0%)	28 (18.7%)	41 (14.6%)	0.336
Metabolic disorders	35 (8.1%)	14 (9.3%)	21 (7.5%)	0.625
Neuromuscular disorder	25 (5.8%)	8 (5.3%)	17 (6.0%)	0.762
Seizure disorder / epilepsy	85 (19.7%)	26 (17.3%)	59 (21.0%)	0.363
Congenital immunodeficiency	8 (1.9%)	4 (2.7%)	4 (1.4%)	0.458
Malignancy with chemoradiotherapy	5 (1.2%)	2 (1.3%)	3 (1.1%)	0.806
Neutropaenia (WBC < 500)	9 (2.1%)	3 (2.0%)	6 (2.1%)	0.926
Solid organ transplant	2 (0.5%)	0 (0.0%)	2 (0.7%)	0.545
Neoplastic disorders	1 (0.2%)	1 (0.7%)	0 (0.0%)	0.348
Haematologic disorders	37 (8.6%)	10 (6.7%)	27 (9.6%)	0.368
**Source of infection**				
Respiratory / pneumonia	246 (57.1%)	86 (57.3%)	160 (56.9%)	0.104
Abdominal / gastroenteritis	44 (10.2%)	14 (9.3%)	30 (10.7%)	
CNS: Meningitis / encephalitis / brain abscess	27 (6.3%)	8 (5.3%)	19 (6.8%)	
Genitourinary infection	25 (5.8%)	16 (10.7%)	9 (3.2%)	
Skin / soft tissue infection	22 (5.1%)	8 (5.3%)	14 (5.0%)	
Primary blood stream infection	14 (3.2%)	5 (3.3%)	9 (3.2%)	
Head, ear, nose, and throat infection	7 (1.6%)	2 (1.3%)	5 (1.8%)	
Osteomyelitis / arthritis	3 (0.7%)	0 (0.0%)	3 (1.1%)	
Others	43 (10.0%)	11 (7.3%)	32 (11.4%)	
**Source of PICU admission**				0.122
ER	296 (68.7%)	97 (64.7%)	199 (70.8%)	
OR/PACU	5 (1.8%)	5 (1.2%)	0	
Outside facility	10 (2.3%)	3 (2%)	7 (2.5%)	
Ward	120 (27.8%)	50 (33.3%)	70 (24.9%)	
Any positive bacterial culture	168 (39.0%)	46 (30.7%)	122 (43.4%)	0.013
Positive viral result	179 (41.5%)	69 (46.0%)	110 (39.1%)	0.203
Positive fungal result	13 (3.0%)	5 (3.3%)	8 (2.8%)	1.0
Paediatric Index of Mortality 3 predicted death rate	8 (2–18)	2 (0–8)	9 (3–18)	< 0.001
PELOD-2 score on day 1	6 (2–11)	2.5 (0–6)	7 (2–14)	< 0.001
PICU length of stay (days)	6 (2–11)	2.5 (0–6)	7 (2–14)	< 0.001
Hospital length of stay (days)	15 (12–31)	12 (6–37)	15 (11–31)	0.043
Mortality	94 (21.8%)	18 (12.0%)	76 (27.0%)	< 0.001

*WBC* white blood cell, *CNS* central nervous system, *PELOD-2* Pediatric Logistic Organ Dysfunction, *PICU* paediatric intensive care unit.

**Table 2:** Performance of the Phoenix Sepsis Score compared with the IPSCC count and PELOD-2 score.

Predictors	AUROC (95% CI)	AUPRC (95% CI)
Phoenix sepsis score	0.67 (0.61, 0.74)	0.45 (0.35, 0.56)
IPSCC count	0.67 (0.60, 0.73)	0.38 (0.29, 0.48)
PELOD-2 score	0.72 (0.65, 0.77)	0.48 (0.37, 0.58)

*IPSCC* International Paediatric Sepsis Consensus Conference, *PELOD-2* Pediatric Logistic Organ Dysfunction-2, *AUROC* area under the receiver-operating characteristic curve, *AUPRC* area under the precision-recall curve, *CI* confidence interval.

## Discussion

In this retrospective analysis of a paediatric critical care sepsis registry, we demonstrated that the PSS had good precision-recall performance and fair discrimination for mortality, supporting its applicability in critical care settings in Saudi Arabia. The score showed higher precision-recall performance than the IPSCC count, with performance comparable to the PELOD-2 score, despite relying on fewer data points across four organ systems. Furthermore, an increased PSS was consistently associated with a higher risk of in-hospital mortality, reinforcing its utility in risk stratification of critically ill children.

The performance of the PSS in our study, including higher precision–recall performance than the IPSCC count are consistent with prior external validation studies^9’15–17^. While the PELOD-2 score showed comparable precision-recall performance and discriminative abilities, it incorporated a broader range of physiological variables across the five organ systems. By contrast, the PSS is based on only four core domains and fewer variables. The score was designed for practicality and broad applicability in both low- and high-resource settings^12,18^. The exclusion of certain organ systems was not due to a lack of clinical relevance but rather a deliberate trade-off for simplicity and operational feasibility. The better performance of the PELOD-2 score in our cohort aligns with the original derivation and validation of the Phoenix criteria as well as that of external validation studies^9’17^.

In our cohort of critically ill paediatric patients admitted to the PICU, the PSS demonstrated fair discriminative ability, with an AUROC of 0.67 (95% CI: 0.61–0.74). Although this value falls below the threshold typically considered indicative of good discrimination, it may reflect the differences in case mix and settings from the original validation study^19^. Our cohort included only patients admitted to a single-centre PICU with suspected sepsis and did not include all patients with infections. The inclusion of only PICU-admitted patients likely resulted in a higher acuity population than those in prior validation studies, which may influence model performance and discrimination. This is supported by the relatively high in-hospital mortality rate (21.8%). These factors may have reduced the discriminative ability of the score. This is consistent with findings from a multicentre UK cohort of critically ill children transported to the PICU, where the PSS also showed a fair discriminative ability with an AUROC of 0.72 (95% CI: 0.56–0.88), highlighting similar limitations in predictive performance among high-acuity PICU populations^20^. Nevertheless, the PSS demonstrated good precision-recall performance and a clear association with increased mortality, supporting its clinical utility even in this high-acuity population.

Mortality did not differ significantly between sepsis and septic shock groups in our cohort, and this finding should be interpreted cautiously. The relatively small number of patients classified as sepsis may have limited statistical power to detect differences in mortality. Our study included only PICU patients with clinically identified sepsis, representing a high-acuity population with substantial baseline mortality risk, which may attenuate differences between groups. Additionally, the PSS incorporates dysfunction across only four organ systems. The reduced separation between sepsis and septic shock categories does not necessarily indicate a limitation of the cardiovascular component’s risk-stratifying capacity but may instead reflect the influence of organ failures not captured by the Phoenix score^21^.

An increased PSS was consistently associated with a higher risk of in-hospital mortality in our cohort, reinforcing its utility for risk stratification in critically ill children. Multiple studies, including the original multicentre development and validation and subsequent external validations, have consistently demonstrated the same association between higher PSS and an increased risk of in-hospital mortality^9’15–17^. These findings across multiple cohorts and healthcare settings underscore the potential of the PSS as a tool for prognostic stratification, in addition to diagnosis.

In our cohort, a PSS threshold of ≥ 2 demonstrated high sensitivity for identifying patients at risk of mortality and a moderate positive predictive value. The higher sensitivity and positive predictive value compared with an emergency department cohort likely reflect the greater illness severity and baseline risk in our PICU population^16^. These findings are consistent with those reported in a single-centre PICU study^15^.

Our study has certain limitations. First, it was conducted in a single tertiary-care PICU, which may limit its generalisability to other settings. Second, unlike the original PSS protocol, our study included only patients admitted to the PICU with clinically identified sepsis rather than all hospitalized patients with infection. This represents a high-acuity, preselected population with greater illness severity and baseline mortality risk, potentially introducing selection bias. Such a restricted case-mix may affect discrimination metrics and limit direct comparison with studies conducted in broader “suspected infection” cohorts. Accordingly, our findings should be interpreted primarily in the context of critically ill PICU populations. Third, inclusion was based on clinical suspicion of infection rather than standardized diagnostic criteria for sepsis, which may introduce heterogeneity in case identification. Additionally, our cohort had a high proportion of children with chronic comorbidities, particularly neurologic conditions, which may have led to an overestimation of sepsis due to baseline abnormalities, such as a low Glasgow Coma Scale score unrelated to the acute presentation. Finally, although the overall sample size was moderate, the relatively small number of mortality events may have limited the precision and stability of performance estimates, particularly the confidence intervals around discrimination metrics.

## Conclusions

In this single-centre study of critically ill paediatric patients in Saudi Arabia, the PSS demonstrated good precision-recall performance and fair discrimination of in-hospital mortality. Its precision-recall performance was comparable to that of the PELOD-2 score despite using fewer variables and organ systems. Importantly, a higher PSS was associated with a higher mortality risk, supporting its value for risk stratification. This study adds to the growing body of evidence supporting the PSS as a reliable tool for mortality risk stratification in paediatric critical care in diverse healthcare settings. However, further external validation in broader and more heterogeneous populations is warranted to confirm its generalisability.

## Supplementary Information

Below is the link to the electronic supplementary material.

Supplementary Material 1

## Data Availability

The datasets generated and/or analyzed during the current study are not publicly available due to privacy restrictions but are available from the corresponding author on reasonable request.

## Abbreviations

AUPRC: Area under the precision-recall curve
AUROC: Area under the receiver-operating characteristic curve
CI: Confidence interval
IPSCC: International Paediatric Sepsis Consensus Conference
IQR: Interquartile range
PELOD-2: Paediatric Logistic Organ Dysfunction-2
PICU: Paediatric intensive care unit
PSS: Phoenix Sepsis Score
SCCM: Society of Critical Care Medicine
SIRS: Systemic inflammatory response syndrome
