# Supplementary material for: External validation of the Phoenix Sepsis Score in a paediatric intensive care unit in Saudi Arabia
Source: Sci Rep. 2026 Apr 14;16:17478. doi: 10.1038/s41598-026-48807-6 (PMC13237370; doi:10.1038/s41598-026-48807-6)
Supplement: Supplementary file 1 — Supplementary Material 1 [file 41598_2026_48807_MOESM1_ESM.docx]

**Article title:** Assessment of the Phoenix Sepsis Criteria in Paediatric Intensive Care: A Retrospective Validation Study in Saudi Arabia

**Journal name**: Scientific Reports

**Author names:**

Yasser Mohammed Kazzaz, MD, FRCPC, MPH¹^,^²^,^³
Naila Shaheen, MD^3,4^
Hamad Alkhalaf, MD¹^,^²^,^³
Ghaida Mashraqi, MD²
Aisha Abdullah Asidan, MD²
Rayouf Mohammed Almojel, MD²
Nawaf Abdullah Alghamdi, MD²
Majd Abdullah Alomar, MD²
Hamza Alali, MD¹^,^²

**Affiliations:**

^1^Department of Paediatrics, Ministry of the National Guard - Health Affairs, Riyadh, Saudi Arabia

^2^ King Saud bin Abdulaziz University for Health Sciences, Riyadh, Saudi Arabia

^3^King Abdullah International Medical Research Centre, King Abdulaziz Medical City, Riyadh, Saudi Arabia

^4^Division of Biostatistics, Department of Population Health, King Abdullah International Medical Research Center, Riyadh, Saudi Arabia

**Corresponding Author**:

Yasser Kazzaz

E-mail: kazzazy@mngha.med.sa

**Keywords:** sepsis; septic shock; paediatric intensive care unit; criteria; Saudi Arabia

**Table of Content**

| **Content** | **Page number** |
| --- | --- |
| Online Resource 1 | 4 |
| Online Resource 2 | 7 |

**Online Resource 1.** Clinical characteristics of critically ill children: Comparison of survivors vs. non-survivors

| Variable | Survivors (N = 337) | Non-survivors (N = 94) | *p*-value |
| --- | --- | --- | --- |
| Male n(%) | 188 (55.79%) | 50 (53.19%) | 0.725 |
| Age (months) median (IQR) | 16 (2–71) | 12 (3.0–57) | 0.705 |
| Any comorbidities n(%) | 258 (76.56%) | 76 (80.85%) | 0.406 |
| Prematurity n(%) | 58 (17.21%) | 11 (11.70%) | 0.265 |
| Metabolic disorder n(%) | 29 (8.61%) | 6 (6.38%) | 0.669 |
| Neuromuscular disorder n(%) | 17 (5.04%) | 8 (8.51%) | 0.214 |
| Seizure disorder / epilepsy n(%) | 66 (19.58%) | 19 (20.21%) | 0.884 |
| Congenital immunodeficiency n(%) | 7 (2.08%) | 1 (1.06%) | 1 |
| Malignancy with chemoradiotherapy n(%) | 4 (1.19%) | 1 (1.06%) | 1 |
| Neutropaenia (WBC < 500) n(%) | 6 (1.78%) | 3 (3.19%) | 0.416 |
| Solid organ transplantation n(%) | 1 (0.30%) | 1 (1.06%) | 0.389 |
| Neoplastic disorder n(%) | 1 (0.30%) | 0 (0.00%) | 1 |
| Haematologic disorder | 27 (8.01%) | 10 (10.64%) | 0.410 |
| Source of infection n(%) |  |  |  |
| Respiratory / pneumonia n(%) | 193 (57.27%) | 53 (56.38%) | 0.367 |
| Abdominal / gastroenteritis n(%) | 34 (10.09%) | 10 (10.64%) |  |
| CNS: Meningitis / encephalitis / brain abscess n(%) | 20 (5.93%) | 7 (7.45%) |  |
| Genitourinary infection n(%) | 20 (5.93%) | 5 (5.32%) |  |
| Skin / soft tissue infection | 18 (5.34%) | 4 (4.26%) |  |
| Primary blood stream infection | 13 (3.86%) | 1 (1.06%) |  |
| Head, ear, nose, and throat | 7 (2.08%) | 0 (0.00%) |  |
| Osteomyelitis / arthritis | 1 (0.30%) | 2 (2.13%) |  |
| Other | 31 (9.20%) | 12 (12.77%) |  |
| Positive bacterial culture | 118 (35.01%) | 50 (53.19%) | 0.002 |
| Positive viral sample | 151 (44.81%) | 28 (29.79%) | 0.009 |
| Positive fungal sample | 6 (1.78%) | 7 (7.45%) | 0.01 |
| Paediatric Index of Mortality 3  predicted death rate median (IQR) | 2 (0–4) | 5 (0–16) | <0.001 |
| Phoenix Score median (IQR) | 2 (1–3) | 4.00 (2–7) | <0.001 |
| PELOD-2 total score 1 median (IQR) | 4 (2–8) | 8.00 (3–12) | <0.001 |
| PICU length of stay (days) median (IQR) | 4 (2–14) | 6.00 (3–12.50) |  |
| Hospital length of stay (days) median (IQR) median (IQR) | 31 (14–40) | 15.00 (6–57) | 0.125 |
| IPSCC score meadin (IQR) | 4.00 (2–14) | 6.00 (3–12.50) | <0.001 |
| CRRT n(%) | 10 (2.97%) | 12 (12.77%) | <0.001 |
| Inotropes n(%) | 112 (33.23%) | 61 (64.89%) | <0.001 |
| Intubation n(%) | 115 (34.12%) | 74 (78.72%) | <0.001 |

WBC, white blood cell count; CNS, central nervous system; IPSCC, International Paediatric Sepsis Consensus Conference; PELOD-2, Paediatric Logistic Organ Dysfunction-2; CRRT, continuous renal replacement therapy; PICU, paediatric intensive care unit.

**Online Resource 2.** Clinical characteristics of critically ill children: Comparison of sepsis vs. septic shock defined by the Phoenix criteria

| Variable | Sepsis (N = 84) | Septic shock (N = 197) | *p-*value |
| --- | --- | --- | --- |
| Male | 45 (53.57%) | 107 (54.31%) | 1 |
| Age (months) | 14 (2–46.5) | 28 (6–99) | 0.036 |
| Any comorbidities | 69 (82.14%) | 149 (75.63%) | 0.231 |
| Prematurity | 10 (11.90%) | 31 (15.74%) | 0.464 |
| Metabolic disorder | 5 (5.95%) | 16 (8.12%) | 0.626 |
| Neurologic - neuromuscular disorder | 7 (8.33%) | 10 (5.08%) | 0.294 |
| Neurologic - seizure disorder / epilepsy | 24 (28.57%) | 35 (17.77%) | 0.042 |
| Immunodeficiency - congenital | 0 (0.00%) | 4 (2.03%) | 0.188 |
| Immunodeficiency - malignancy with chemoradiotherapy | 0 (0.00%) | 3 (1.52%) | 0.255 |
| Immunodeficiency - Neutropaenia (WBC < 500) | 1 (1.19%) | 5 (2.54%) | 0.474 |
| Immunodeficiency - solid organ transplantation | 0 (0.00%) | 2 (1.02%) | 0.354 |
| Haematologic disorder | 4 (4.76%) | 23 (11.68%) | 0.072 |
| Source of infection |  |  |  |
| Respiratory / pneumonia | 46 (54.76%) | 114 (57.87%) | 0.752 |
| Abdominal / gastroenteritis | 12 (14.29%) | 18 (9.14%) |  |
| CNS: Meningitis / encephalitis / brain abscess | 7 (8.33%) | 12 (6.09%) |  |
| Genitourinary infection | 4 (4.76%) | 5 (2.54%) |  |
| Skin / soft tissue infection | 4 (4.76%) | 10 (5.08%) |  |
| Primary blood stream infection | 2 (2.38%) | 7 (3.55%) |  |
| Head, ear, nose, and throat | 1 (1.19%) | 4 (2.03%) |  |
| Osteomyelitis / arthritis | 0 (0.00%) | 3 (1.52%) |  |
| Others | 8 (9.52%) | 24 (12.18%) |  |
| Positive bacterial culture | 36 (42.86%) | 86 (43.65%) | 0.902 |
| Positive viral result | 36 (42.86%) | 74 (37.56%) | 0.425 |
| Positive fungal result | 1 (1.19%) | 7 (3.55%) | 0.443 |
| Paediatric Index of Mortality 3  predicted death rate | 7 (2–16) | 10 (3–18) | 0.033 |
| PELOD-2 score day 1 | 5.85 (3.50–6.00) | 8.18 (6.00–9.00) | <0.001 |
| PICU length of stay (days) | 13.27 (7–12) | 8.86 (5–9) | <0.001 |
| Hospital length of stay (days) | 60.12 (23–49.50) | 54.65 (13–23) | <0.001 |
| Mortality | 20 (23.81%) | 56 (28.43%) | 0.4251 |

WBC, white blood cell count; CNS, central nervous system; PELOD-2, Paediatric Logistic Organ Dysfunction-2; PICU, paediatric intensive care unit.
